# Cardiac magnetic resonance in Pompe disease: a systematic literature review

**DOI:** 10.1007/s11547-026-02199-9

**Published:** 2026-04-25

**Authors:** Amalia Lupi, Elisa Schiavone, Vincenza Gragnaniello, Sara Cervaro, Filippo Crimì, Emilio Quaia, Alberto Burlina, Alessia Pepe

**Affiliations:** 1 Department of Medicine – DIMED, Institute of Radiology, University of Padua, Padua, Italy; 2 Division of Inherited Metabolic Diseases, Department of Women’s and Children’s Health, University of Padua, Padua, Italy

**Keywords:** Pompe disease, Magnetic resonance imaging, Rare disease, Cardiovascular abnormality

## Abstract

Pompe disease (PD) is a rare genetic disorder, caused by the lysosomal acid alpha-glucosidase enzyme deficiency leading to accumulation of glycogen, primarily in skeletal and respiratory muscles, but also in the heart. In both known PD forms, namely infantile (IOPD) and late-onset (LOPD), the prevalence and nature of myocardial involvement are still to be clarified. Cardiovascular magnetic resonance (CMR) may help to detect unrecognized alterations contributing to patients’ management. This study aimed to systematically review PD CMR features. We conducted a systematic search of three electronic databases (PubMed, Scopus, and Web of Science) up to February 2024, without language or time interval restriction. Two reviewers performed the search and selection process, data extraction, and synthesis. We resolved disagreements by consensus and/or involving a third reviewer. The included studies have been classified according to the Oxford Centre for Evidence Based Medicine (CEBM) grading system. Out of 276 articles, 11 papers were finally included: seven about IOPD and four regarding LOPD. According to CEBM, seven were level 5, one was level 4, two were level 3, and one was level 2. Six studies reported qualitative late-gadolinium enhancement tissue characterization, 1 described cardiac infiltration on TIRM sequences, 3 explored changes in ExtraCellular Volume and, among these, 2 evaluated also T1/T2 mapping and perfusion; one study assessed the management after therapy through T1 mapping. Literature data regarding CMR features in PD are limited and based on nonparametric assessment mainly. Further investigations, involving also mapping techniques, are required to better understand PD cardiac involvement.

## Introduction

Pompe disease (PD), OMIM #232300, is an autosomal recessive lysosomal storage disorder caused by the deficient activity of the lysosomal acid alpha-glucosidase (GAA) enzyme. This results in impaired glycogen degradation and accumulation within the lysosomes [1].

Clinically, PD includes a broad spectrum of phenotypes, ranging from a rapidly progressive infantile form (IOPD) to a slowly progressive childhood/adult late-onset form (LOPD). Classic IOPD manifests soon after birth and is characterized by absent or nearly absent GAA activity, severe muscle weakness, cardiomyopathy, and respiratory insufficiency, which typically lead to death within the first year of life in the absence of enzyme replacement therapy (ERT) [2, 3]. Contrariwise, LOPD comprises milder subtypes in which the disease manifests later in childhood, adolescence, or adulthood, with slowly progressive disease, characterized by skeletal and respiratory muscle weakness, that is frequently the leading cause of death [3, 4]. While clinically relevant cardiomyopathy is uncommon in this form, the prevalence and nature of myocardial abnormalities are still to be clarified also for addressing the optimal timing to start ERT, particularly in adolescents and adults with late-onset PD disease [5–7].

Cardiovascular magnetic resonance (CMR) is the gold-standard technique for assessing cardiac volumes, mass, and function, and for non-invasive tissue characterization [8]. In addition, parametric mapping techniques, which allow precise and quantitative assessment of the myocardium, are significantly changing the management of patients with cardiac involvement. Therefore, according to the literature, a deeper understanding of cardiac involvement in PD patients through CMR could reveal additional diagnostic and prognostic value [9].

The aim of this systematic literature review (SLR) was to summarize existing evidence on CMR findings in both infantile and late-onset Pompe disease and to identify knowledge gaps to guide future research.

## Materials and methods

We performed a comprehensive literature search in three major electronic databases (PubMed, Scopus, and Web of Science) up to February 7, 2024, without applying any restrictions on language or publication date. The search strategy combined terms related to Pompe Disease (Pompe disease, alpha-glucosidase deficiency, glycogen storage disorder) with those pertaining to cardiac MR (Cardiovascular Magnetic Resonance, Cardiac Magnetic Resonance Imaging, Magnetic Resonance), by adding MeSH terms and synonyms (e.g., Glycogen Storage Disease Type II, Acid Maltase Deficiency, Pompe Disease, cardi* MRI/CMR) and applying field tags and truncations.

The full search string was (“Pompe Disease”[Mesh] OR "Glycogen Storage Disease Type II"[Mesh] OR "Pompe disease"[tiab] OR "Glycogen Storage Disease Type II"[tiab] OR "GSD II"[tiab] OR "Acid Maltase Deficiency"[tiab] OR "alpha-glucosidase deficiency"[tiab] OR "glycogen storage disorder*"[tiab]) AND ("Magnetic Resonance Imaging"[Mesh] OR "Cardiac Imaging Techniques"[Mesh] OR "Cardiovascular Magnetic Resonance"[tiab] OR "Cardiac Magnetic Resonance"[tiab] OR "CMR"[tiab] OR "cardi* MRI"[tiab] OR "cardi* MR"[tiab] OR "Magnetic Resonance"[tiab] OR "MRI"[tiab]).

Duplicate records were automatically removed using Endnote (Clarivate). Two reviewers (ES and AL) independently screened all titles and abstracts of the identified records. Any record judged potentially eligible by either reviewer was retrieved in full and assessed independently. Disagreements were resolved through third-reviewer (AP) arbitration.

The inclusion criteria were:

- studies directly addressing the role of cardiac MR in patients with Pompe disease;
- original research articles, cohort studies, case series, and case reports.

The exclusion criteria were:

- studies not involving CMR assessment;
- articles not focused on Pompe disease;
- reviews, editorials, conference abstracts without full text, letters, and non-original content;
- animal studies or in vitro studies;
- papers lacking sufficient imaging data to allow extraction.

In addition, a weekly automated email alert was configured to capture newly published studies from the included databases that were screened independently as described above according to inclusion criteria.

Data extraction was performed independently by two investigators (ES and AL). Study characteristics, such as authors, publication year, journal, country, study design, sample size, age of participants, PD form (LOPD or IOPD), CMR techniques and findings, were independently collected.

The primary outcomes were LGE prevalence/pattern, hypertrophic phenotype, mapping/ECV; secondary outcomes were perfusion deficits, feature tracking, LA size. Missed data are specified.

The two reviewers then compared their extractions and worked together to resolve any differences in order to obtain a single summary table collecting all the information and ensuring data accuracy. A third reviewer was finally involved to avoid misunderstandings.

The Oxford centre for evidence-based medicine (CEBM) grading system was used to determine the level of evidence for the data extracted from the full texts included in the study [10].

Results were grouped for PD form (IOPD and LOPD) and therapy regimen (ERT or not). Descriptive statistics were applied to extracted variables using SPSS Software (version 24).

Moreover, a structured risk of bias (RoB) assessment was carried out for all included studies by two reviewers independently (AP and AL, controversies resolved by agreement) and following:

- JBI (Joanna Briggs Institute) tools for the case reports [11];
- NOS (Newcastle–Ottawa Scale) checklist for the cohort/cross-sectional studies [12].

Sample description, data collection procedures and synthesis criteria, as well as RoB assessment accomplished standards defined by PRISMA 2020 guidelines [13, 14].

## Results

**Fig. 1:**
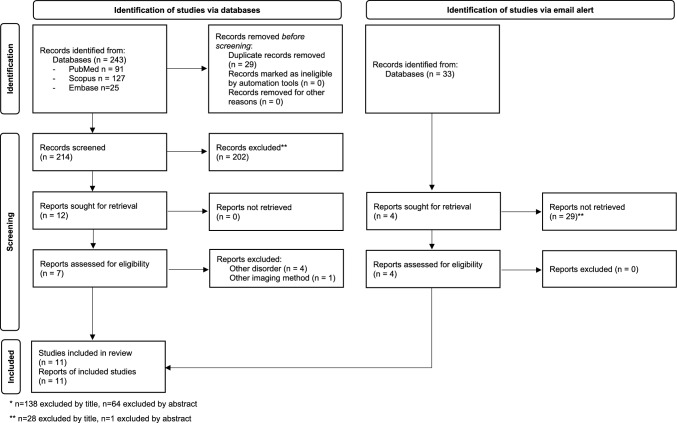
PRISMA 2020 flow diagram for systematic review showing records’ selection

The search strategy identified a total of 276 records, among these 33 via email alert. The last update was received on November 17, 2025. After screening titles and abstracts, 16 full-text articles were reviewed for eligibility. Among these, eleven papers were included (Table 1) [6, 15–24]. A complete PRISMA 2020 flow diagram is included as Fig. 1, with detailed counts at each stage and exclusion reasons. Five full-text articles were excluded because they did not match the inclusion criteria [25–29]: in particular, four out of five articles were about other glycogen storage disorders, and in the other study CMR evaluation was not reported.

**Table 1:** Characteristics of the included studies

Author	Journal	Year	Country	LoE	Sample (n)	Age	PD form	CMR techniques
Barker et al	Molecular Genetic and Metabolism	2010	USA	3	10	1–38 months (range)	IOPD	SSFP cine imaging for function; IRSS SSFP or segmented GE for LGE
Boentert et al	Journal of Cardiovascular Magnetic Resonance	2016	Germany	2	17	50 ± 18 years (mean ± SD)	LOPD	SSFP cine imaging for function; IR gradient echo sequences for LGE; MOLLI sequences for ECV imaging
Boxer et al	Journal of Computer-Assisted Tomography	1986	USA	5	1	5 months	IOPD	spin-echo pulse sequences
Gragnaniello et al	Journal of Clinical Medicine	2023	Italy	5	1	3.5 years	IOPD	Long and short-axis cine-SSFP
Hussein et al	Italian Journal of Pediatrics	2025	Egypt	4	8	7.43 ± 2.7 months	IOPD	SSFP cine sequence, LGE, T1-T2 mapping, ECV, tissue tracking and phase contrast
Lasam et al	Cureus	2025	USA	5	1	58 years	LOPD	na
Morris et al	International Journal of Cardiovascular Imaging	2015	Germany	3	12 (4 CMR)	37.9 ± 13.4 years (mean ± SD)	LOPD	LGE
Walczak-Galezewska et al	European Review for Medical and Pharmacological Sciences	2017	Poland	5	1	54 years	LOPD	4-chamber and 2-chamber TIRM sequences
Wang et al	JACC: Case Reports	2024	China	5	1	1 year	IOPD	Cine sequence, T1 mapping (sequences not reported)
Zhong et al	Heliyon	2023	China	5	1	9 months	IOPD	SSTSE and SSFP for function, PSIR for LGE
Zhu et al	European Heart Journal—Cardiovascular Imaging	2025	China	5	1	3 months	IOPD	Cine sequences, perfusion, LGE sequences, T1 and T2 mapping, ECV (sequences not reported)

*LoE* level of evidence, *PD* Pompe Disease, *IOPD* infantile-onset Pompe Disease, *LOPD* late-onset Pompe Disease, *CMR* cardiovascular magnetic resonance, *SSFP* steady state free precession, *IRSS* inversion recovery single shot, *GE* gadolinium enhancement, *LGE* late gadolinium enhancement, *IR* inversion recovery, *MOLLI* modified look-locker inversion recovery, *ECV* extracellular volume, *MR* magnetic resonance, *TIRM* turbo inversion recovery magnitude, *SSTSE* steady-state turbo spin echo, *PSIR* phase sensitive inversion recovery, *LVMI* left ventricle mass index, *LA* left atrium, *LVEF* left ventricle ejection fraction, *Na* not available

All 11 included articles were written in English and published between 1986 and 2025 [6, 15–24]. Three studies were conducted in the USA [15, 17, 20], two in Germany [6, 16], three in China [22–24], one in Italy [18], one in Poland [21], and one in Egypt [19]. Regarding the level of evidence, one was level 2 [16], two were level 3 [6, 15], one was level 4 [19], and seven were level 5 [17, 18, 20–24] (Table 1).

Among the 11 papers included in the review, 7 (64%) were about IOPD [15, 17–19, 22–24], and 4 (36%) were about LOPD [6, 16, 20, 21]. In four studies (36.4%), three about IOPD [15, 18, 19] and one about LOPD [16], patients were on ERT; in one (9%) case report, the patient was on gene therapy [22]; in 3 studies (27.3%) patients were untreated [20, 21, 24], while in the remaining 3 studies (27.3%) therapy was not reported [6, 17, 23].

All studies investigated for alterations in cardiac function and morphology; furthermore, out of the 11 studies, 6 reported late-gadolinium enhancement (LGE) data [6, 15, 16, 19, 23, 24], one study described cardiac infiltration on turbo inversion recovery magnitude (TIRM) T2-weighted sequences [21], one study evaluated T1 mapping before and after gene therapy [22], 3 studies explored changes in extracellular volume (ECV) [16, 19, 24] and 2 of them explored also cardiac perfusion, T1/T2 mapping, and/or feature tracking [19, 24] (Table 2).

**Table 2:** Primary outcomes stratified by IOPD/LOPD

Study (year)	PD form	ERT/GT (yes/no)	Sample n	LGE: n /*N* (%)	LGE pattern (brief)	Hypertrophic phenotype: n/*N* (%)	Hypertrophy metrics reported (wall thickness/LVMI)	Mapping/ ECV abnormality: *n*/*N* (%)	Mapping/ECV details
IOPD studies (individual)									
Barker et al., 2010	IOPD	Yes (ERT)	10	**1/10 (10.0%)**	Anterior & antero-lateral mid-basal (*n* = 1)	**10/ 10 (100.0%)**	No significant change in LVMI 94.0 (range 43.8–334.0) vs 44.5 (range 34.0–303.0) *p* = 0.44 (data on 5 FU)	**NR/10**	–
Boxer et al., 1986	IOPD	NR	1	**NR/1**	–	**1/1 (100%)**	Right, left, and septal hypertrophy (no thickness values)	**NR/1**	–
Gragnaniello et al., 2023	IOPD	Yes (ERT)	1	**NR/1**	–	**0/1 (0%)**	Remodeling from hypertrophic to non-compaction phenotype (NC/C ratios 4/4.1) after ERT	**NR/1**	_
Hussein et al., 2025	IOPD	Yes (ERT)	8	**8/8 (100%)**	2/8 with marked LGE at aortic root	**8/ 8**	Elevated LVMI (116.2 ± 14.02) compared to normal reference values (65.2 ± 1.9, *p* = 0.000)	**8/8 (100%)**	Compared to normal reference values, global T2 (mean 65 ± 4.3 ms vs 56.5 ± 1.61, *p* = 0.000) significantly elevated, ECV no significantly elevated (mean 33.5 ± 15.3% vs 28.2 ± 0.8%, p = 0.42). No data on T1 mapping
Wang et al., 2024	IOPD	Yes (GT)	1	**NR/1**	–	**1/1 (100%)**	Severe ventricular and septal thickening, reduced after ERT (no millimeters provided). LVEF 89% vs 86%	**1/1 (100%)**	After GT, reduction in native T1 values (1,351.69 ms vs 1,294.22 ms in mid anteroseptal wall, 1,273.31 ms vs 1,218.17 ms at the insertion site). No normal reference values reported
Zhong et al., 2023	IOPD	NR	1	**1/1 (100%)**	Bi-ventricular “halo-like” diffuse LGE	**1/1 (100%)**	Bi-ventricular and septal hypertrophy (no numeric thickness)	**NR/1**	_
Zhu et al., 2025	IOPD	no	1	**1/1 (100%)**	Septum mid-wall patchy enhancement	**1/1 (100%)**	Bilateral concentric myocardial and papillary muscle hypertrophy; LVEF 68%	**1/1 (100%)**	Increased native T1 (1265 ms), T2 (40 ms) and ECV 34% in affected region. No normal reference values reported
IOPD—pooled (from above IOPD studies)	**IOPD (pooled)**	**ERT in 19; GT in 1, NR in 2, no in 1**	**23**	**11/20 (55%)**	Patterns heterogeneous non ischemic: focal mid-wall inferolateral, diffuse “halo-like”, mid-wall patchy	**22/23 (96%)**	Hypertrophic phenotype in all patients except one patient with NC remodeling after ERT	**10/10 (100%)**	↑T1/T2/ECV
LOPD studies (individual)									
Boentert et al., 2016	LOPD	yes (in 12)	17	**3/17 (17.6%)**	Mid-wall basal inferolateral LGE (*n* = 3)	**1/17 (5.9%)**	Mild septal hypertrophy (*n* = 1)	**3/ 14 (21%)**	Increased ECV in 3 patients, no significant difference in ECV between patients and controls (27.3 ± 4.5% vs 25.4 ± 2.5, *p* = 0.18). ECV by using MOLLI sequences, not successfully acquired in 3 patients
Lasam et al., 2025	LOPD	no	1	**NR/1**	_	**1/1 (100%)**	Hypertrophic cardiomyopathy with LVOT flow restriction (no numeric thickness)	**NR/1**	_
Morris et al., 2015	LOPD	NR	4	**0/4 (0%)**	–	**0/4 (0%)**	–	**NR/4**	_
Walczak-Galezewska et al., 2017	LOPD	no	1	**NR/ 1**	_	**NR/1**	_	**NR/1**	_
LOPD—pooled (from above LOPD studies)	**LOPD (pooled)**	**ERT in 12**	**23**	**3/21 (14%)**	Non ischemic pattern (mid-wall basal inferolateral)	**2/22 (9%)**	mild septal hypertrophy (Boentert 1/17) and single case HCM (Lasam 1/1)	**3/14 (21%)**	17/31 no mapping data

*NR* not reported

In 2 case reports of patients with IOPD, cardiac biventricular and septal hypertrophy was found [17, 23]; in another case report, a 58-year-old woman with a gradual onset of exertional dyspnea and fatigue showed hypertrophic phenotype cardiomyopathy (HCM) with left ventricular outflow tract (LVOT) obstruction on CMR, and a LOPD was then diagnosed [20]. Zhong et al. [23] also investigated for LGE, reporting a biventricular and septal multifocal, patchy pattern.

Gragnaniello et al. [18] followed LV mass indexed to body surface area (LVMI) and ejection fraction (EF) by echocardiography in a pediatric IOPD patient before and after the administration of two different types of ERT and then CMR confirmed the remodeling from hypertrophic to NC phenotype.

Barker et al. [15] evaluated 10 IOPD patients on ERT by both echocardiography and CMR, assessing LVMI and EF at baseline, and in half of the patients, they assessed changes in LVMI and EF, showing no statistically significant differences over time.

In Morris et al. [6] study, 12 LOPD patients underwent morphological and functional cardiac assessment by echocardiography, and CMR performed in a subgroup of patients (*n* = 4) showed no myocardial structural alterations nor LGE. Similarly, in the cohort study by Boentert et al. [16] patients had normal ventricular volumes, EF, and systolic function based on feature tracking analysis, but 71% of them had been receiving ERT; out of 17 patients, 3 had basal inferior-lateral LGE and two were also hypertensive and demonstrated high global ECV values in addition to dilated left atrium. However, no significant differences were observed between patients and controls.

Wang et al. [22] reported a reduction in T1 mapping values after gene therapy in an IOPD 1-year-old child. Zhu et al. [24] reported instead an increase of T1, T2, and ECV values in a septal mid-wall area characterized by patchy enhancement on LGE technique, in an IOPD 3-month-old patient with biventricular hypertrophy and hypokinesis; a left ventricular linear basal subendocardial perfusion defect was also described.

In another recent study, 8 Egyptian IOPD patients underwent clinical, genetic, and laboratory evaluation, as well as CMR 6 months after starting ERT administration: all patients showed alterations detected by LGE technique and affection of all feature-tracking parameters, as well as an increase in global T2 value and in ECV fraction [19].

Finally, a case report of a 54-year-old sportsman who presented with syncope described “multiple storage materials” detected by echocardiography, and hyperintensity on CMR TIRM T2 sequences interpreted as infiltration; thus, in the suspicion of accumulation pathology, LOPD was diagnosed even if no genetic diagnosis is reported [21].

Primary outcomes (LGE prevalence and pattern, hypertrophic phenotype (wall thickness/LVMI), mapping/ECV abnormalities) stratified by IOPD/LOPD and ERT status are reported in Table 2. Across the pooled dataset, LGE was significantly more prevalent in IOPD (55%, 11/20; 95% CI 0.34–0.74) than LOPD (14%, 3/21; 95% CI 0.05–0.35). Hypertrophic phenotype was present in 96% of IOPD patients (22/23; 95% CI 0.79–0.99) versus 9% in LOPD (2/22; 95% CI 0.03–0.28). Mapping and ECV abnormalities were also substantially more frequent in IOPD, observed in 100% (10/10; 95% CI 0.72–1.00), while only 21% of LOPD patients (3/14; 95% CI 0.08–0.48) demonstrated abnormal tissue characteristics. Overall, the pooled analysis demonstrates a consistently higher burden of structural and tissue-level myocardial involvement in IOPD relative to LOPD across all CMR markers.

Full RoB tables are provided (Tables 3 and 4). Overall, the studies displayed variable methodological rigor, reflecting differences in study design, sample size, and detail of CMR reporting. Based on JBI assessment for case reports, all studies reported demographic findings and current clinical conditions on presentation; with the exception of one study [23], diagnostic CMR methods were unclear or not described, producing moderate–to-high risk of bias. Based on NOS scoring for cohort/cross-sectional studies [6, 15, 16, 19], the selection category achieved 3–4/4 stars, and comparability 1–2/2; the high quality/low risk of bias was achieved only for cohort studies.

**Table 3:** JBI assessment—case reports

Study (single-case)	1	2	3	4	5	6	7	8	Overall judgment
Boxer et al., 1986	**Yes**	**Unclear**	**Yes**	**No** (early CMR spin-echo, minimal protocol)	**No**	**NA**	**No**	**Unclear**	**High risk** (information limited)
Gragnaniello et al., 2023	**Yes**	**Yes**	**Yes**	**Unclear** (NC/C ratio was calculated in no standard way on SA views)	**Yes**	**Yes**	**Yes**	**Yes**	**Moderate** (limited CMR data with methodological drawbacks)
Lasam et al., 2025	**Yes**	**Yes**	**Yes**	**No** (No description about the CMR methodology)	**Yes**	**Yes**	**Unclear**	**Unclear**	**High risk** (CMR techniques NR)
Walczak-Galezewska et al., 2017	**Yes**	**Yes**	**Yes**	**No** (only generic description of CMR clinical findings, only TIRM images without description)	**No**	**NA**	**No**	**Unclear**	**High risk** (CMR methodology no described and incomplete)
Wang et al., 2024	**Yes**	**Yes**	**Yes**	**Unclear** (no detail about the sequences and no normal reference values reported for mapping)	**Yes**	**Yes**	**Unclear**	**Yes**	**Moderate** (sequence reporting incomplete and no normal reference values)
Zhong et al., 2023	**Yes**	**Yes**	**Yes**	**Yes**	**Unclear**	**NA**	**No**	**Yes**	**Moderate risk** (CMR limited to non-parametric techniques)
Zhu et al., 2025	**Yes**	**Yes**	**Yes**	**Unclear**	**Yes**	**Yes**	**Yes**	**Yes**	**Moderate risk** (advanced mapping reported without normal reference values)

1. Patient demographic reported? 2. Patient history and timeline? 3. Current clinical condition on presentation? 4. Diagnostic tests/assessment methods described? 5. Intervention(s) (if any) described? 6. Post-intervention clinical condition/outcome reported? 7. Adverse events described? 8. Takeaway/lessons for practice?

**Table 4:** NOS scoring—cohort/cross-sectional studies

Study	Selection (0–4)		Comparability (0–2)		Outcome (0–3)		Total stars /9	Short rationale
Barker et al., 2010	**3/4—**no selection of a comparison group		**1/2—**limited adjustment for confounders		**1/3—**FU in 5/10 patients limited to imaging data		**5/9**	Moderate quality/Moderate risk of bias (FU in 5/10 patients limited to imaging data)
Boentert et al., 2016	**4/4**		**2/2—**limited adjustment for key confounders (ERT duration)		**3/3**		**9/9**	High quality/Low risk of bias (limited control for key confounders)
Hussein et al., 2025	**3/4—**no selection of a comparison group		**1/2—**limited adjustment for confounders		**0/3—**cross-sectional		**4/9**	Moderate quality/Moderate risk of bias (Cross-sectional study without a selection of a comparison group and limited adjustment for confounders)
Morris et al., 2015	**4/4**		**2/2**		**3/3**		**9/9**	High quality/Low risk of bias

## Discussion

PD is a severe metabolic myopathy whose symptoms are caused by pathogenic variants in the gene encoding acid alpha-glucosidase (GAA). Lack of the enzyme leads to lysosomal glycogen accumulation in many tissues. However, in PD, symptoms and dysfunction mainly affect the heart and skeletal muscle [30], and the clinical course depends mainly on the age of the onset, the type of pathogenic variants, and the resulting level of residual GAA activity [31]. In this clinical scenario, knowledge of the disease may increase substantially through the application of new imaging and molecular techniques.

This SLR aims to explore current evidence about the role of CMR in PD. Our results show that in the literature only a few studies about CMR findings in PD were performed. Moreover, most of the papers had a retrospective study design, small sample sizes, and therefore only a low/moderate level of evidence. Worldwide prevalence of this rare disease is unknown, but 2 large studies based on data provided by newborn screening (NBS) reported similar birth prevalence and incidence (respectively 1/18,711 and 1/18,795) [32, 33]. Nowadays, thanks to NBS, early diagnosis of such rare diseases is possible, allowing for better therapeutic management. The main current therapy is enzyme replacement therapy (ERT) with recombinant human GAA (rhGAA), which has changed the natural history of this disease, especially in IOPD [34].

In our review, only five studies included patients on ERT/gene therapy [15, 16, 18, 19, 22]. Among these, Barker et al. [15] reported no significant variations in LVMI and EF by CMR, unlike what was observed in the case report by Gragnaniello et al. [18], and confirmed by other literature studies that show significant decreases in LVMI evaluated by echocardiography [35, 36].

Moreover, even if our results show that CMR findings in LOPD patients seem to be milder or absent, and non-specific [6, 16, 20, 21], these are based on limited and nonparametric sequences; indeed, only one study applied advanced feature tracking techniques and ECV assessment [16]. Among IOPD studies, only one reported a reduction in native T1 values 4 months post-gene therapy [22]; another study found elevated T1, T2, and ECV values in a septal area characterized by LGE [24]. Unfortunately, both works are strongly limited by the lack of reporting the center-specific reference values, and it is well known that parametric mapping techniques need center-specific cut-off [37, 38].

In most of the included articles, CMR evaluation followed echocardiographic assessment [6, 15, 17–21, 24], even though CMR allows for a more precise wall thickness and mass evaluation, also in follow-up and comparison between patients, and provides additional information about tissue characterization. Nowadays, CMR is the gold-standard technique for detecting non-invasively cardiac involvement by quantitative functional and tissue characterizations. This technique is significantly changing the patients’ management, improving diagnostic accuracy and quantitative follow-up in order to tailor the therapy [8]. Nevertheless, the main advantages associated with CMR are the non-invasive tissue characterization and the non-ionizing nature of this technique, which makes it very attractive, especially in the pediatric population. For these reasons, despite the paucity of literature data on PD patients, based on the recent last guidelines for the management of cardiomyopathies, contrast-enhanced CMR is recommended in class I (level of evidence B) in any patients with cardiomyopathy at initial evaluation, in class IIa (level of evidence C) during the follow-up to monitor the disease progression and to aid the risk stratification and management, and in class IIb (level of evidence C) in genotype-positive/phenotype-negative family members to aid diagnosis and detection of early cardiac involvement to tailor the therapy [9]. However, it should not be overlooked that CMR has intrinsic limits regarding primarily the duration of investigations and the sedation-related risk in PD patients [39], but the overall diagnostic value of CMR should be considered [40].

LGE prevalence differed markedly by phenotype: IOPD pooled 55% (11/20) vs LOPD pooled 14% (3/21) in the available dataset, suggesting LGE is more commonly present in infantile-onset cases. Patterns varied from focal mid-wall inferolateral LGE to diffuse “halo-like” involvement in infants. Hypertrophic phenotype was reported more often in IOPD (22/23, 96%) than LOPD (2/22, 9%) in this sample; however, numerical thickness or LVMI values were infrequently reported (LVMI change reported only in Barker et al.). Mapping/ECV abnormalities were substantially more frequently documented in IOPD patients (10/10, 100%) than LOPD (3/14, 21%), likely reflecting disease biology. In particular, Hussein et al. (2025) reported mapping abnormalities in their whole cohort (8/8), compared to normal reference values.

Heterogeneous reporting about ERT effects prevents robust subgroup inference.

Moreover, in addition, few studies used advanced parametric mapping techniques.

From the RoB assessment, a moderate/high level of heterogeneity and potential bias emerged in most of the studies. Although all case report studies reported demographic findings and current clinical conditions on presentation, in almost all diagnostic CMR methods were unclear or not described, producing a moderate–to-high risk of bias (e.g., no mapping site-specific reference values, no sequence parameters or vendor details, NC/C ratio calculated in no standard way on SA view, inappropriate infiltration evaluation by TIRM T2 sequences).

The cross-sectional studies (15,19) achieved moderate quality/moderate risk of bias due to no outcome data, related to the nature of the studies, and some selection and comparability limits.

Only in the more robust cohort studies [6, 16], control for confounding factors was good and high quality/low risk of bias was achieved thanks to clear patient selection procedures, full comparability, and outcome data.

Despite these limitations, the collective evidence provides meaningful insights into characteristic CMR patterns in Pompe disease when interpreted within the context of methodological constraints.

Thus, effort should be devoted to performing further research on this topic, aiming to address the diagnostic and prognostic role of advanced contrast-enhanced parametric CMR for managing and tailoring the response to therapy in patients with PD, as it has been done for patients with other rare cardiomyopathies such as amyloidosis, Anderson–Fabry disease, hemochromatosis, and inflammatory cardiomyopathies [41, 42].

In conclusion, our systematic review demonstrates that CMR may provide early cardiac involvement in patients with PD, but few data are available about the possible advantage of a quantitative functional and tissue characterization, rigorously applied in such population. Thus, it is challenging to give new evidence concerning the diagnostic accuracy and the prognostic role of CMR in the management of PD patients, being available literature mostly based on case reports or small cross-sectional studies. This makes it difficult to draw generalized conclusions or clinical implications, actually motivating our effort to delineate the current state of the literature—with the aim of stimulating scientific interest and encouraging a more structured diagnostic work-up in this specific patient population looking for CMR biomarkers. The limited level of evidence available in the current literature clarifies that the findings should be interpreted cautiously and mainly serve to highlight gaps in knowledge.

Precisely for this reason, our intent was not to provide generalized conclusions but to map existing evidence as a foundation for future research rather than clinical recommendations, to standardize the diagnostic work-up in this rare population.

## Other information

Due to the rarity of the topic and the scarcity of the literature, the review was not registered, nor was a protocol prepared. No financial support was received for the review. The authors have no competing interests to disclose.

## Abbreviations

PD: Pompe disease
GAA: Glucosidase alpha acid
IOPD: Infantile-onset Pompe disease
LOPD: Late-onset Pompe disease
CMR: Cardiovascular magnetic resonance
LGE: Late-gadolinium enhancement
ECV: Extra-cellular volume
